# Supplementary material for: Protein-based tools for the detection and characterisation of Oropouche virus infection
Source: EMBO Mol Med. 2025 Aug 11;17(9):2462–82. doi: 10.1038/s44321-025-00291-7 (PMC12423313; doi:10.1038/s44321-025-00291-7)
Supplement: Supplementary file 1 — Table EV1 [file 44321_2025_291_MOESM1_ESM.docx]

**Table EV1. Nanobody sequences.** The complementarity determining region (CDR) of the unique nanobody (VHH) sequences isolated by phage display are shown, with selected nanobodies highlighted in **bold**.

| Antigen | Sequence id | CDR1 | CDR2 | CDR3 |
| --- | --- | --- | --- | --- |
| Gc Spike | **A7** | **GRTFSAYV** | **IRRSYGST** | **AAIRQGGGWGSSAVDY** |
|  | **A9** | **GFTFSTLA** | **INRSGGIV** | **AARTGYAYVRVSDYDS** |
|  | **B6** | **GRTFSNYA** | **LSRSGTST** | **AADPLFQNAAYHY** |
|  | **C6** | **GRTFSRYA** | **ITWSGGNT** | **AAGTQTGRIRDKGPSY** |
|  | **C7** | **GRTFSPNA** | **ISWSGGST** | **AADLEGMVVADRI** |
|  | **E8** | **GRAFSTYA** | **ITLGSRIT** | **AARGQGSPPLRNRWSADDYNY** |
|  | A6 | GRTDSTYA | ISWNGGIT | AADRTAAKVRTGSPPY |
|  | B7 | GINIRGTL | IGRAGES | SAYGNWNTY |
|  | C4 | GRTFSPNA | ISWSGGTT | APDLXGMVLCHRI |
|  | D10 | GRTTSSRA | IRPAGTFT | AAAEGPTVFGRVNDYTY |
|  | E7 | GSFFGIFA | FTWSGVDR | AADLEGTVVGDRI |
|  | E9 | GRTFSNSA | FTRSGAP | GGTASGIXLSRPSDYDY |
|  | F8 | GRTFGTYV | ITWSSGST | AAGEAGRGYGY |
|  | G7 | GRTFSTYA | ITRGGRIT | APGGQGSPPFRNRWSADDYNY |
|  | G9 | GRTFGSYA | FMRSGAP | AGAASRILLSRPSDFDY |
| N | **A2** | **GGIFSFNA** | **IANDGNT** | **SALRRGAQD** |
|  | **D2** | **GFTLDYYS** | **ISSGGST** | **AADIMSCPGKYSAVADY** |
|  | **E2** | **SGFTPNPA** | **IYHGDIT** | **TKGDS** |
|  | **E3** | **GFTFSNAY** | **IYYGDMT** | **TKGDE** |
|  | **F2** | **GRTFSSFP** | **INWSGASK** | **AAGSGRYGYTFTKEYTY** |
|  | **G1** | **GRTFTDYP** | **INWSGGAT** | **AGGSGRLSYNMDKEYIY** |
|  | A12 | GLAFTEYS | MNWSGGKT | AASIQSWDTQYPLAYTY |
|  | B1 | ETNLSSNG | ITSGGTS | YMRSTWSASDY |
|  | C2 | LGTDNVKP | INPSGAI | YARAGVPY |
|  | D3 | GRTFSNYG | INWSGVTT | AAYSTGSYWMESRYDY |
|  | E1 | GRAFSRYT | INWS.SGNT | AASIQNWDTQYPQAYEY |
|  | G3 | ASALSDYA | ISRSGGIT | AARGFERRWRDEALYAY |
|  | H1 | GFIFSDHA | ITTDGTVT | QRAIDDGS |
|  | H3 | GRTLSNYY | ITWDGRAT | AAGSRFGISRSANHYEY |
