# Supplementary figures and images for: Protein-based tools for the detection and characterisation of Oropouche virus infection
Source: EMBO Mol Med. 2025 Aug 11;17(9):2462–82. doi: 10.1038/s44321-025-00291-7 (PMC12423313; doi:10.1038/s44321-025-00291-7)

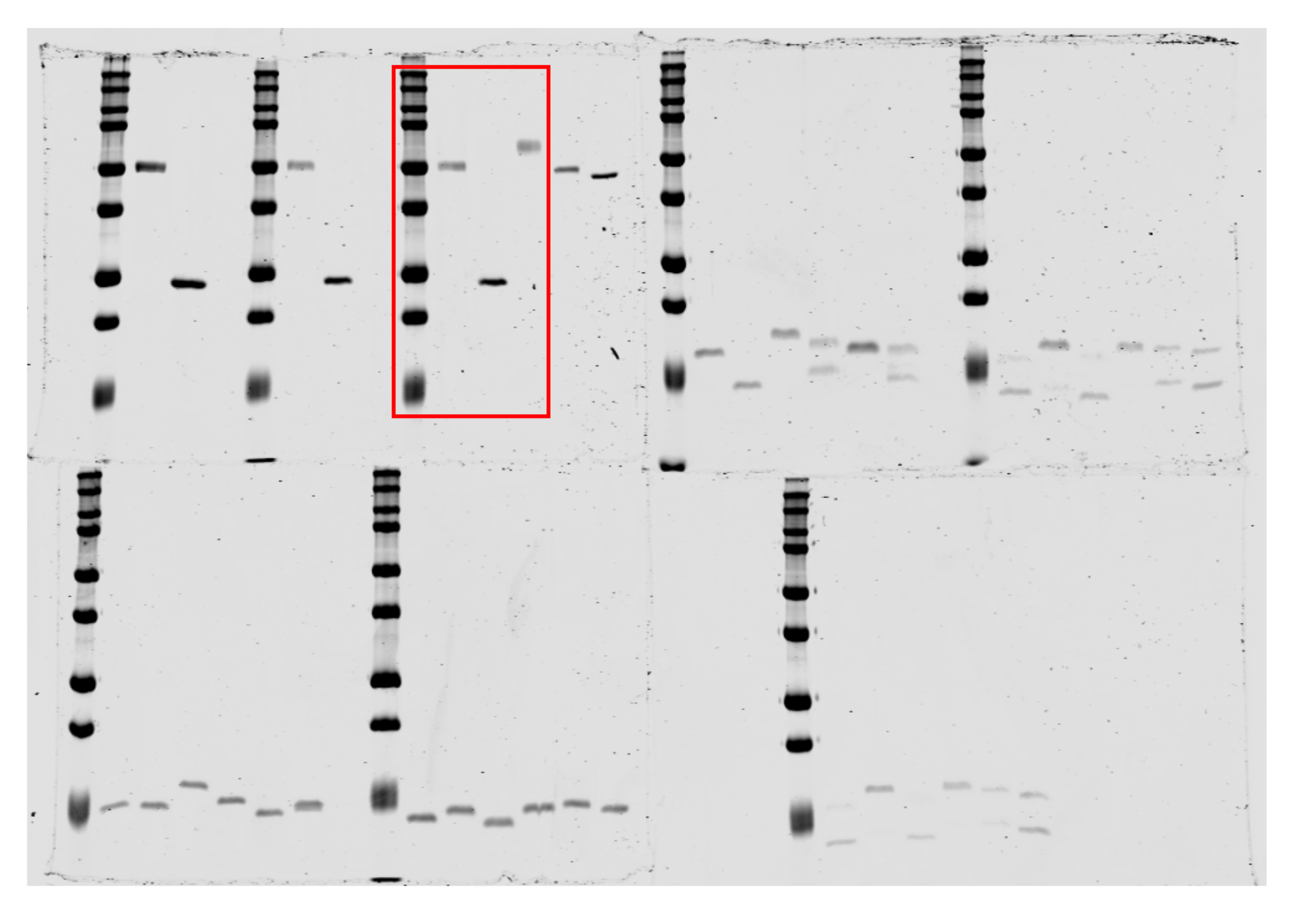

Supplement: Supplementary file 3 — Source data Fig. 1 [file 44321_2025_291_MOESM3_ESM.zip › Figure 1/1B/SDS-PAGE_crop_marks.png]

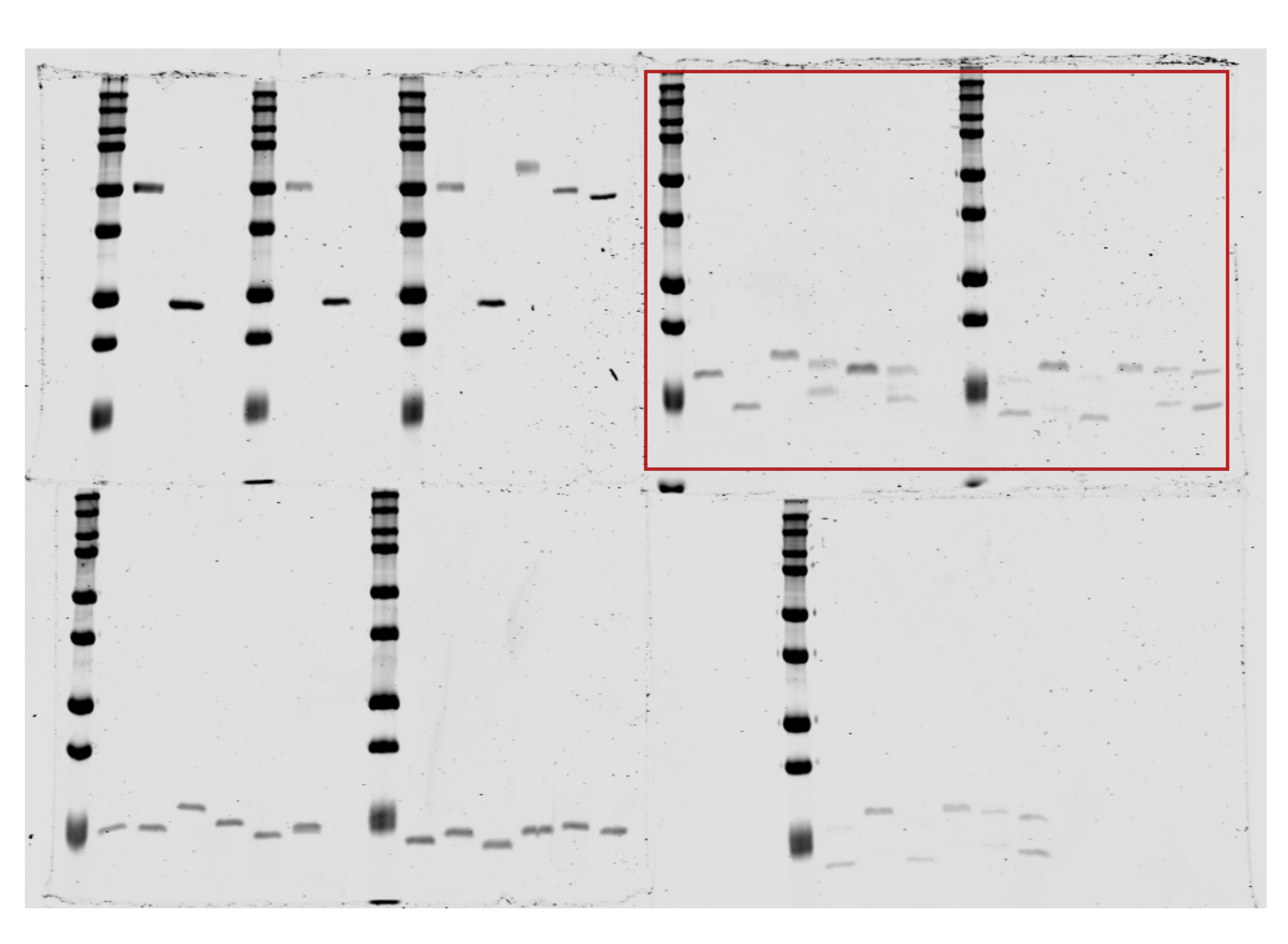

Supplement: Supplementary file 7 — Figure EV2 Source Data [file 44321_2025_291_MOESM7_ESM.zip › Figure EV2/EV2B/EV2B.png]

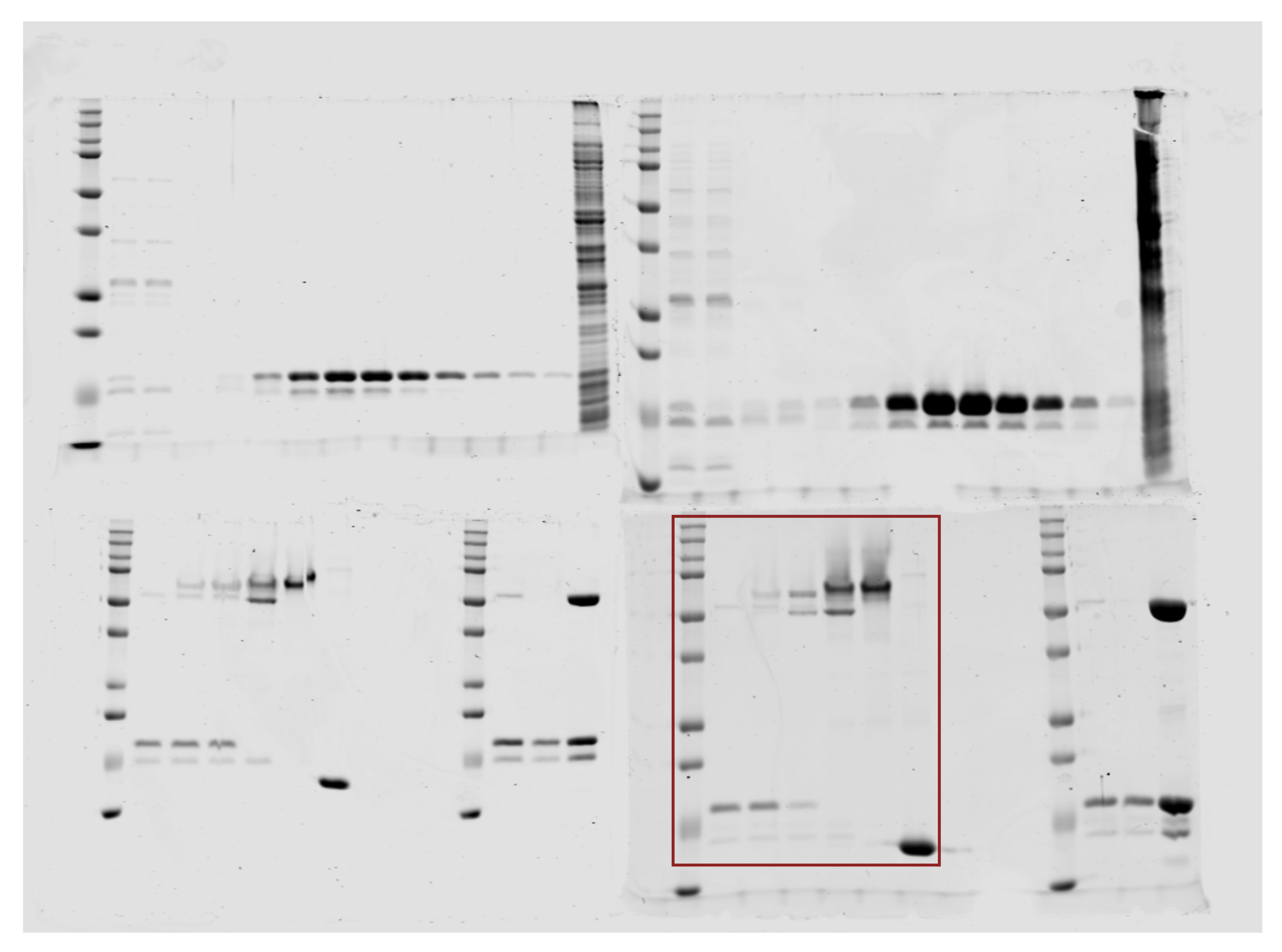

Supplement: Supplementary file 7 — Figure EV2 Source Data [file 44321_2025_291_MOESM7_ESM.zip › Figure EV2/EV2C/EV2C.png]

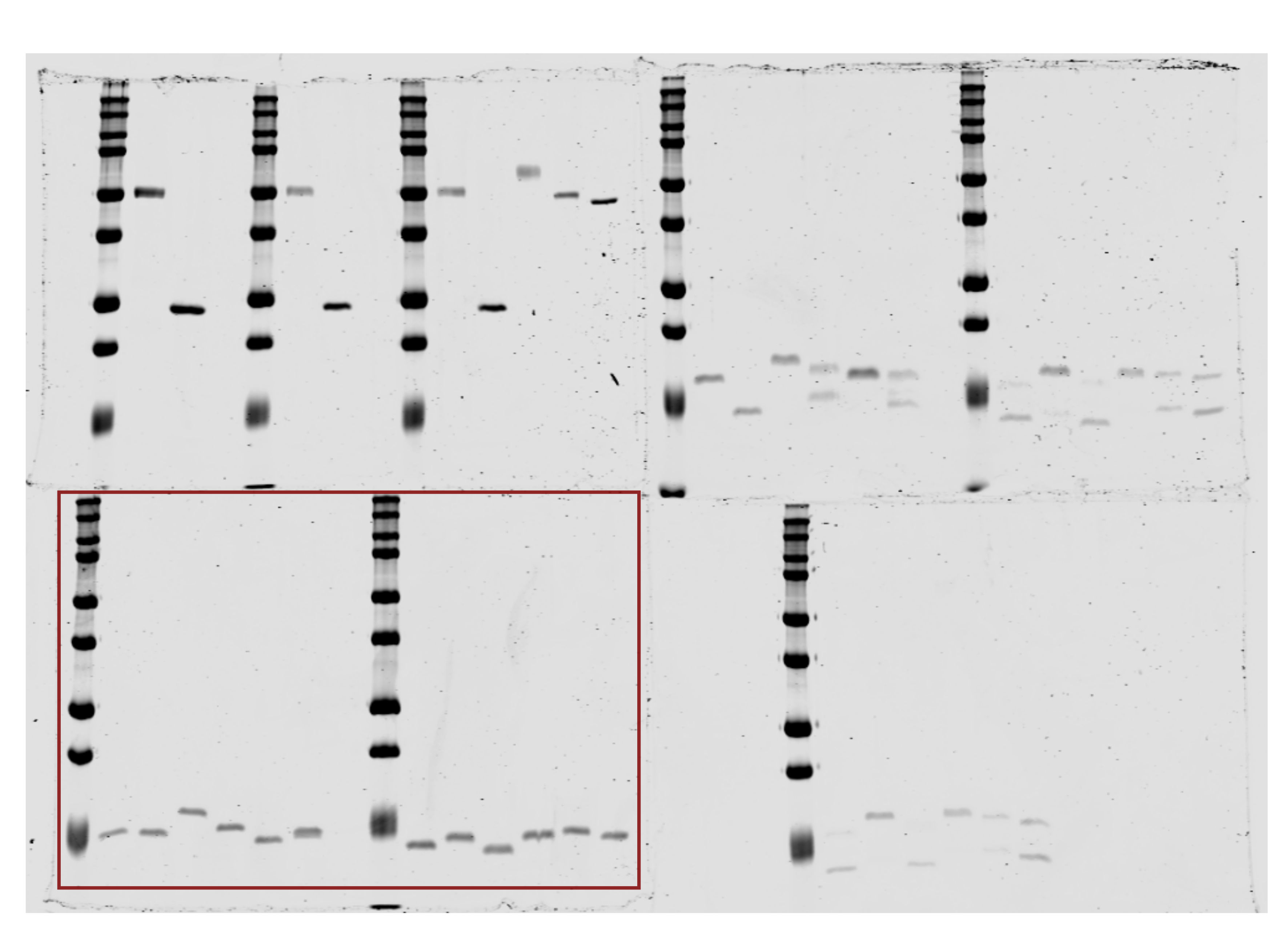

Supplement: Supplementary file 7 — Figure EV2 Source Data [file 44321_2025_291_MOESM7_ESM.zip › Figure EV2/EV2D/EV2D.png]
