# Supplementary material for: Protein-based tools for the detection and characterisation of Oropouche virus infection
Source: EMBO Mol Med. 2025 Aug 11;17(9):2462–82. doi: 10.1038/s44321-025-00291-7 (PMC12423313; doi:10.1038/s44321-025-00291-7)
Supplement: Supplementary file 6 — Source data Fig. 4 [file 44321_2025_291_MOESM6_ESM.zip › Figure 4/4A/Western blot Fig4A.pptx]

## Slide 1
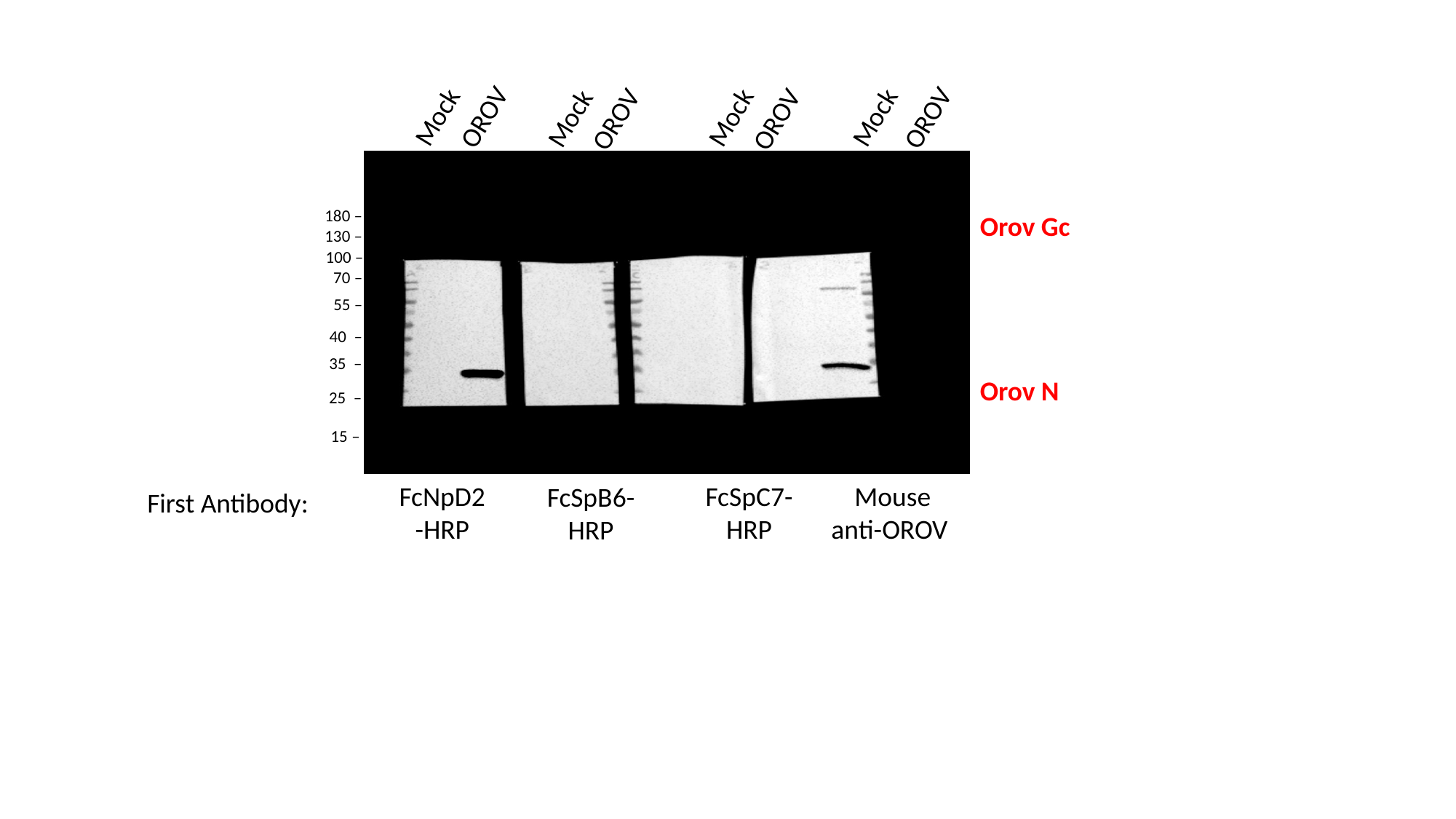

OROV
Mock
Mock
Mock
Mock
OROV
OROV
OROV
180 –
130 –
Orov Gc
100 –
 70 –
55 –
40 –
35 –
Orov N
25 –
15 –
FcNpD2-HRP
FcSpC7-HRP
Mouse anti-OROV
FcSpB6-HRP
First Antibody:

## Slide 2
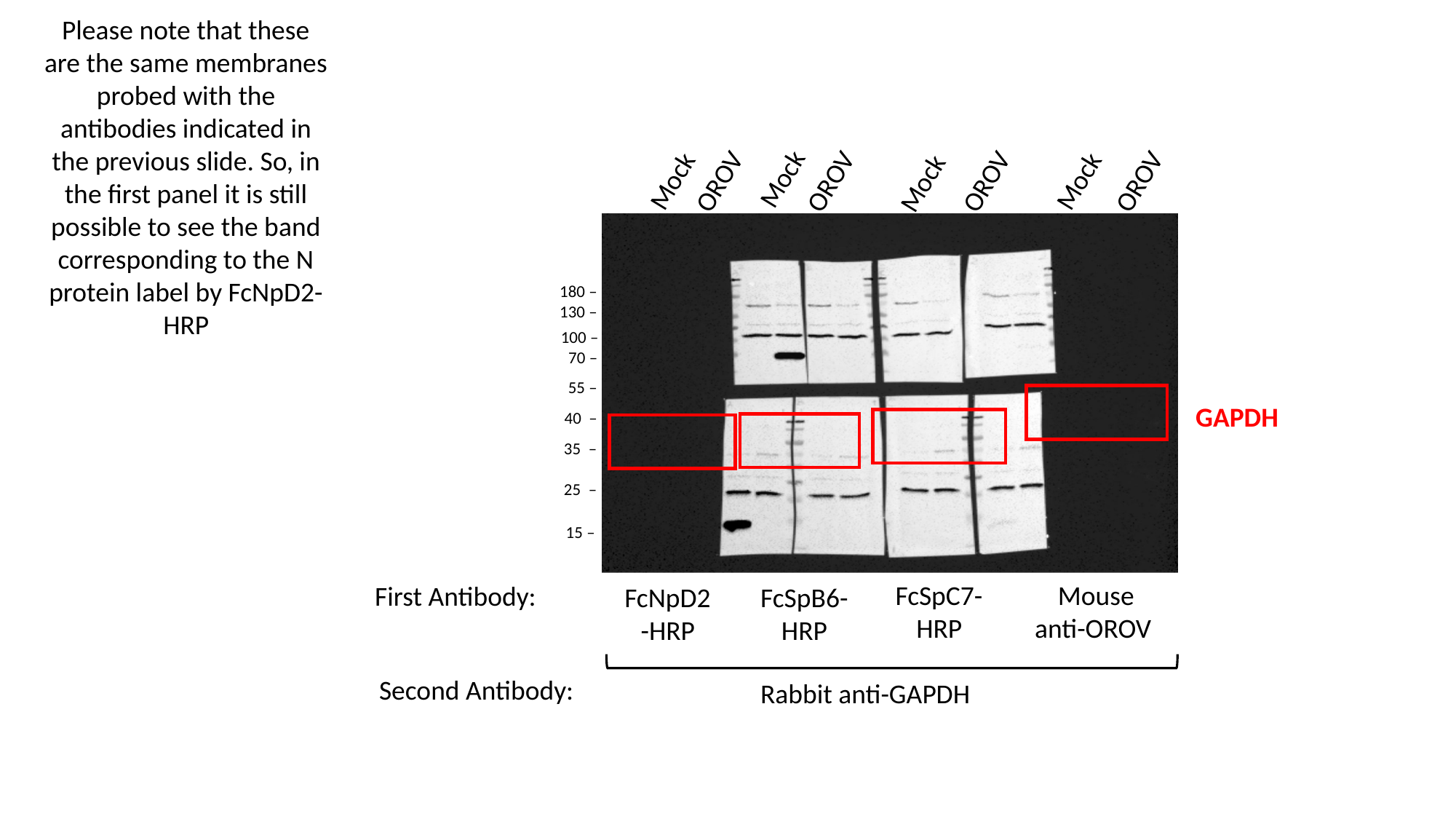

Please note that these are the same membranes probed with the antibodies indicated in the previous slide. So, in the first panel it is still possible to see the band corresponding to the N protein label by FcNpD2-HRP
OROV
Mock
Mock
Mock
Mock
OROV
OROV
OROV
180 –
130 –
100 –
 70 –
55 –
GAPDH
40 –
35 –
25 –
15 –
FcSpC7-HRP
Mouse anti-OROV
First Antibody:
FcNpD2-HRP
FcSpB6-HRP
Second Antibody:
Rabbit anti-GAPDH
