# Supplementary material for: Protein-based tools for the detection and characterisation of Oropouche virus infection
Source: EMBO Mol Med. 2025 Aug 11;17(9):2462–82. doi: 10.1038/s44321-025-00291-7 (PMC12423313; doi:10.1038/s44321-025-00291-7)
Supplement: Supplementary file 8 — Expanded View Figures [file 44321_2025_291_MOESM8_ESM.pdf]

Expanded View Figures

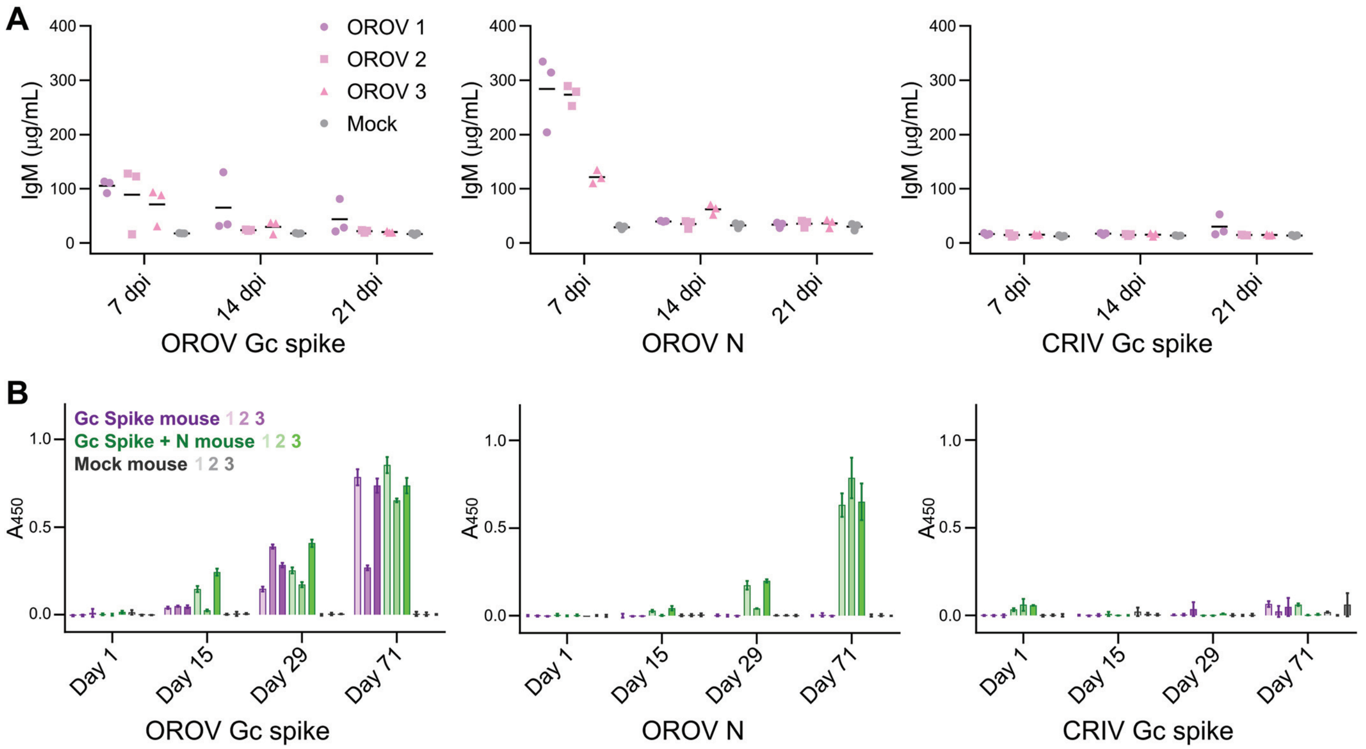

Figure EV1. Purified OROV antigens are recognised by antibodies raised in response to OROV infection, and purified antigens raise an immune response in mice.

(**A**) Blood was harvested at indicated days post-infection (dpi) and an indirect ELISA to detect IgM was performed using indicated antigens. Values are show for three independent ELISA experiments using serum from three infected mice or one mock-infected control mouse. (**B**) Pre-boost serum was collected from mice immunised at day 1 and the boosted at days 15 and 29 with purified OROV antigens Gc spike alone (purple), OROV Gc spike plus N (green), or mock-immunised (3 mice per treatment). Serum was also collected 6 weeks after the final boost. The presence of antibodies that recognise OROV Gc spike, OROV N, or CRIV Gc spike (negative control) was tested by indirect ELISA. Mean ± SD of three independent measurements for each mouse is shown.

© The Author(s)

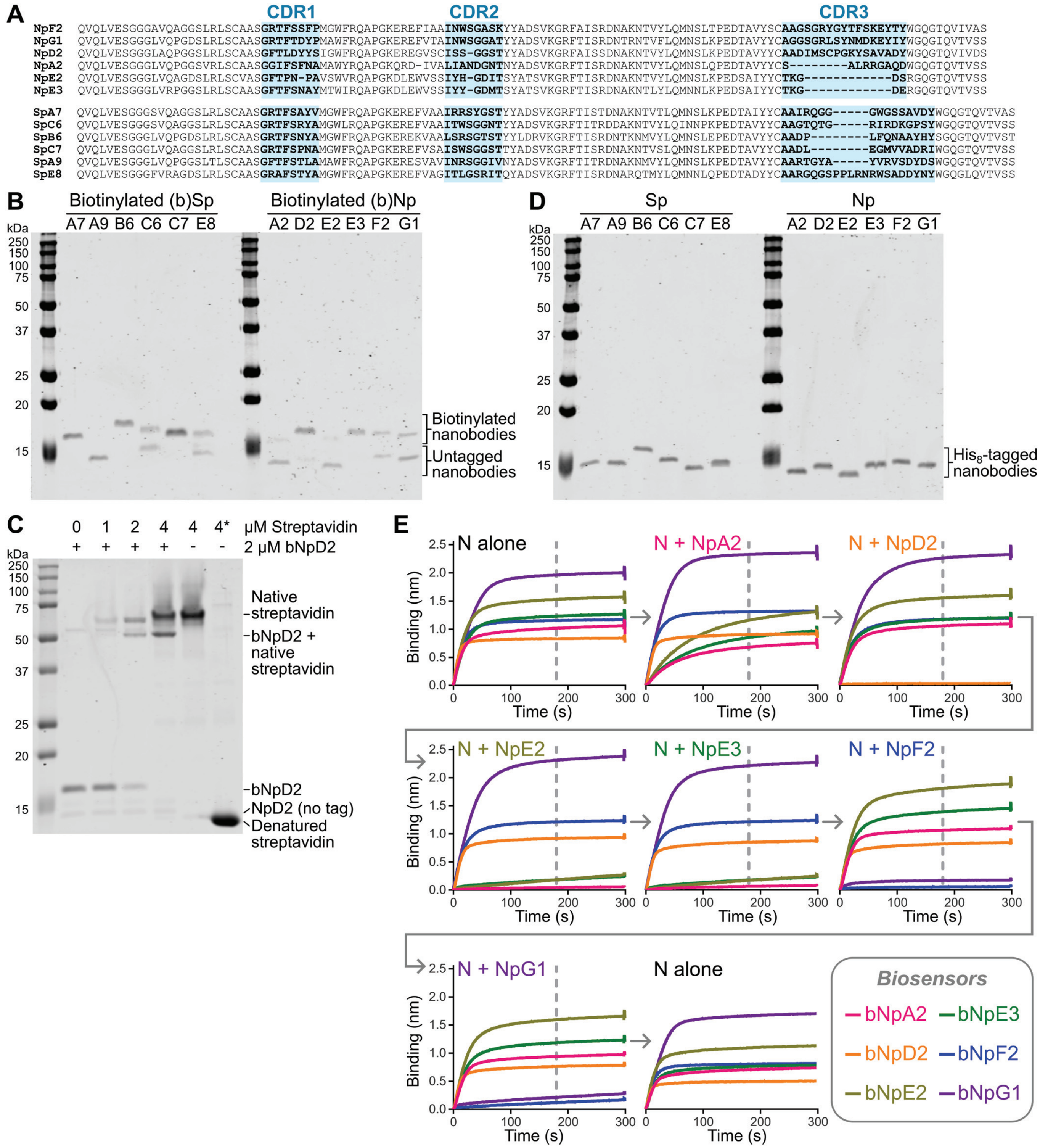
A
CDR1
CDR2
CDR3
NpF2 QVQLVESGGGAVQAGGSLRLSCAASGRTFSSFPMGWFRQAPGKEREFIAAINWSGASKYYADSVKGRFAISRDNAKNTVYLQMNSLTPEDTAVYSCAAGSGRYGYTFSKEYTYWGQGTQVIVAS
NpG1 QVQLVESGGGLVQPGDSLRLSCAASGRTFTDYPMAWFRQAPGKEREFVTAINWSGGATYYADSVKGRFTISRDNTRNTVFLQMNSLKPEDTAVYYCAGGSGRLSYNMDKEYIYWGQGIQVTVSS
NpD2 QVQLVESGGGLAQPGGSLRLSCAASGFTLDYYSIGWFRQAPGKEREGVSCISS-GGSTYYADSVKGRFAISSDNAKNTVYLQMNSLKPEDTAVYYCAADIMSCPGKYSAVADYWGQGTQVIVDS
NpA2 QVQLVESGGGLVQPGGSVRLSCAASGGIFSFNAMAWYRQAPGKQRD-IVALIANDGNTNYADSVKGRFTISRDNAKNTVYLQMNSLQPEDTAVYYCS--------ALRRGAQDWGQGTQVTVSS
NpE2 QVQLVESGGGLVQAGDSLRLSCVASGFTPN-PAVSWVRQAPGKDLEWVSSIYH-GDITSYATSVKGRFTISRDNAKNTVYLQMNSLKPEDSAIYYCTKG------------DSRGQGTQVTVSS
NpE3 QVQLVESGGGLVRPGGSLRLSCAASGFTFSNAYMTWIRQAPGKDLEWVSSIYY-GDMTSYATSVKGRFTISRDNAKNTLYLQMNNLKPEDSAIYYCTKG------------DERGQGTQVTVSS
SpA7 QVQLVESGGGSVQAGGSLRLSCAASGRTFSAYVMGWFRQAPGKEREFVAAIRRSYGSTYYADSVKGRFTISTDNAKNTVTLQMNSLKPEDTAIYYCAAIRQGG-----GWGSSAVDYWGQGTQVTVAS
SpC6 QVQLVESGGGSVQAGGSLRLSCAASGRTFSRYAMGWLRQAPGKEREFVAAITWSGGNTYYADSVKGRFTITRDDAKNTVYLQINNPKPEDTAVYYCAAGTQTG-----RIRDKGPSYWGQGTQVTVSS
SpB6 QVQLVESGGGLVQPGDSLRLSCAASGRTFSNYAMGWFRQAPGKEREKVAALSRSGTSTYYLDRVKGRFTISRDNTKNMVYLQMNSLEPEDTAVYYCAADP--------LFQNAAYHYSGQGTQVTVST
SpC7 QVQLVESGGGLVQAGGSLRLSCAASGRTFSPNAMGWFRQAPGKEREFVSAISWSGGSTTYADSVKGRFTISRDNAKNTVSLQMNSLKPEDTAVYYCAADL--------EGMVVADRIWGQGTQVTVSS
SpA9 QVQLVESGGGLVQPGGSLTLSCAASGFTFSTLAMAWFRQAPGKERESVAVINRSGGIVNYADSVKGRFTITRDNAKNMVYLQMNSLKPEDTAVYYCAARTGYA-----YVRVSDYDSWGQGTQVTVSS
SpE8 QVQLVESGGGFVRAGDSLRLSCAASGRAFSTYAMGWFRQAPGKEREFVAGITLGSRITQYADSVKGRFTISRDNARQTMYLQMNNLQPEDTADYYCAARGQGSPPLRNRWSADDYNYWGQGLQVTVSS
B
Biotinylated (b)Sp
A7 A9 B6 C6 C7 E8
Biotinylated (b)Np
A2 D2 E2 E3 F2 G1
kDa
250
150
100
75
50
37
25
20
15
Biotinylated nanobodies
Untagged nanobodies
D
Sp
A7 A9 B6 C6 C7 E8
Np
A2 D2 E2 E3 F2 G1
kDa
250
150
100
75
50
37
25
20
15
His8-tagged nanobodies
C
0 1 2 4 4 4* μM Streptavidin
+ + + + - - 2 μM bNpD2
kDa
250
150
100
75
50
37
25
20
15
Native streptavidin
bNpD2 + native streptavidin
bNpD2
NpD2 (no tag)
Denatured streptavidin
E
N alone
N + NpA2
N + NpD2
N + NpE2
N + NpE3
N + NpF2
N + NpG1
N alone
Binding (nm)
Time (s)
0.0
0.5
1.0
1.5
2.0
2.5
0
100
200
300
Biosensors
bNpA2
bNpE3
bNpD2
bNpF2
bNpE2
bNpG1

Figure EV2. Generation and purification of nanobodies to detect OROV Gc spike and N.

(**A**) Amino acid sequence alignment of selected nanobodies that recognise OROV N (NpXX) or Gc spike (SpXX). Complementarity Determining Regions (CDRs) as defined by IGMT (Giudicelli et al, 2011) are highlighted. (**B**) Coomassie-stained SDS-PAGE of nanobodies that had been biotinylated in vitro. Upper bands represent the Avi-tagged, biotinylated nanobodies while lower bands represent nanobodies where the tag had been lost, presumably by proteolysis during the purification procedure. (**C**) Electrophoretic mobility shift assay to confirm biotinylation. After boiling of the nanobody in SDS-PAGE loading buffer, streptavidin was added at a 2:1, 1:1 or 1:2 molar ratio (nanobody:streptavidin) and samples were subjected to SDS-PAGE. Appearance of a high apparent molecular weight band, and disappearance of the low molecular weight band, confirms biotinylation of the nanobody. Asterisk (*) denotes streptavidin that was boiled before SDS-PAGE, rather than being added to the sample buffer after boiling. (**D**) Coomassie-stained SDS-PAGE of His_8-tagged nanobodies. (**E**) OROV N competition BLI sensorgrams. Streptavidin biosensors loaded with bNbs were sequentially incubated with 1 µM OROV N then with 1 µM OROV N plus 25 µM of each competitive nanobody, with biosensor regeneration between incubation (association) cycles. After the final competition step, the biosensor was incubated with 1 µM OROV N to confirm that the bNbs remained active. For each association in the presence of competitive nanobodies, the response for each sensor at 180 s (dotted grey line) was divided by the response of the same sensor in the presence of OROV N alone at 180 s to generate the heatmap shown in Fig. 2C. Source data are available online for this figure.

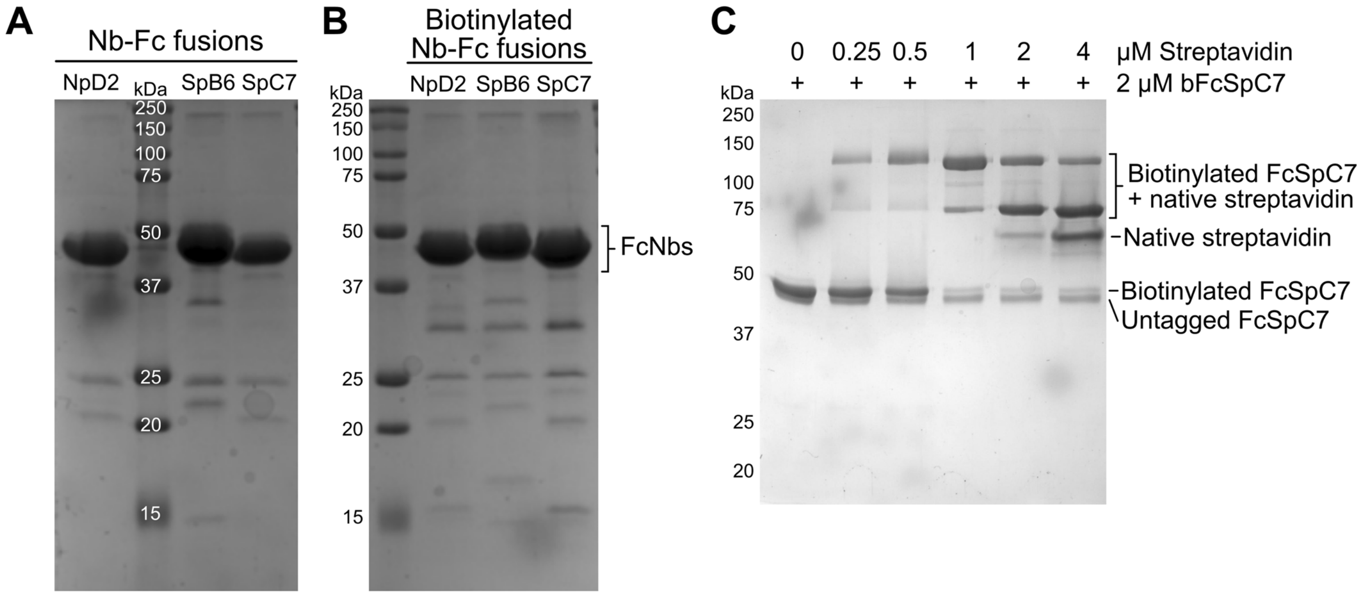

Figure EV3. Purification of nanobody-Fc fusions.

(**A**, **B**) Coomassie-stained SDS-PAGE of (**A**) untagged nanobody-Fc fusions, and (**B**) biotinylated nanobody-Fc fusions. (**C**) Electrophoretic mobility shift assay to confirm biotinylation. After boiling of the nanobody-Fc fusion in SDS-PAGE loading buffer, streptavidin was added at a 8:1, 4:1, 2:1, 1:1 or 1:2 molar ratio (nanobody-Fc fusion:streptavidin) and samples were subjected to SDS-PAGE. Appearance of a high apparent molecular weight bands, and reduction of the lower molecular weight band, confirms biotinylation of the nanobody-Fc fusion.

 © The Author(s)

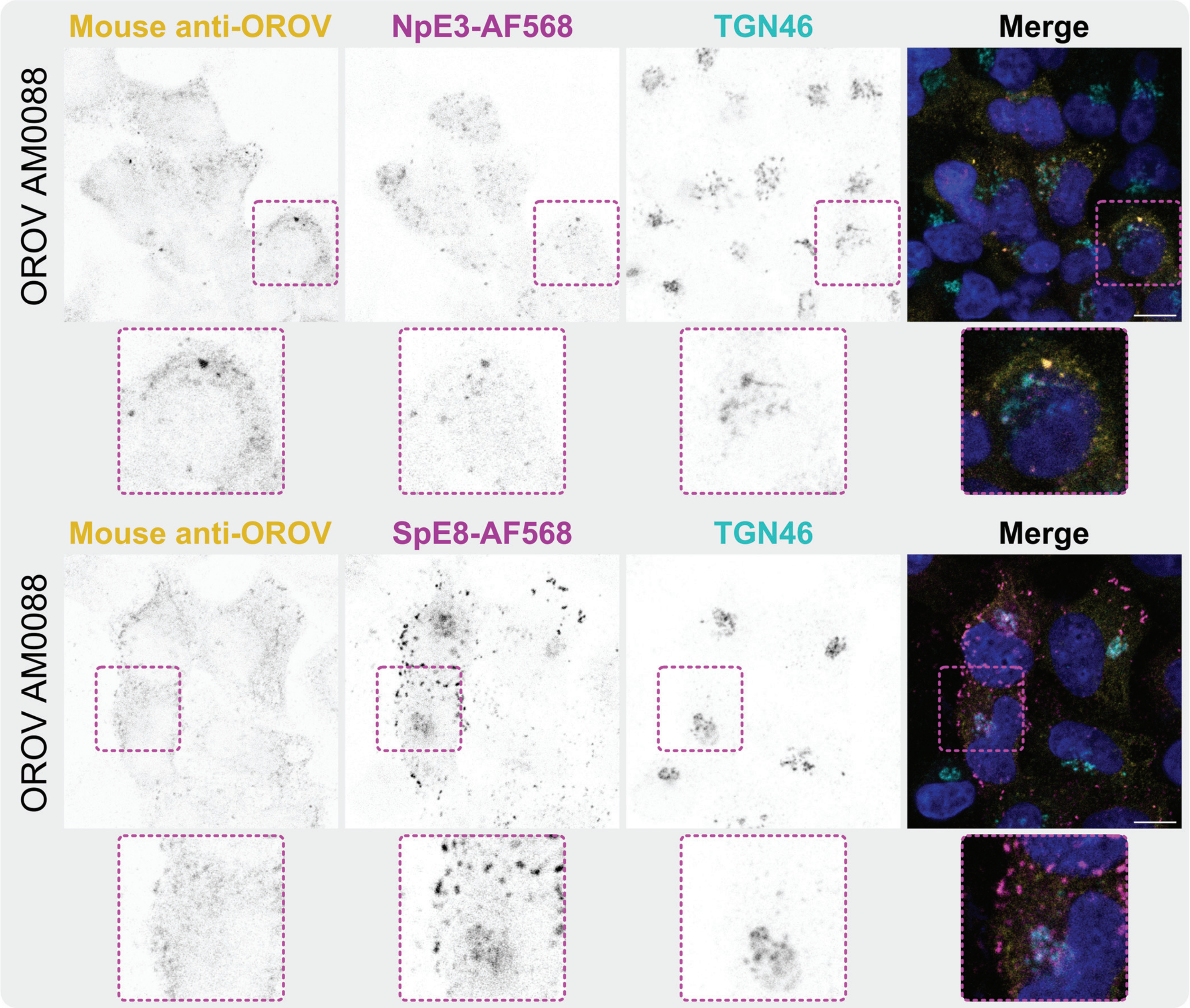

Figure EV4. Nanobodies detect infection of HeLa cells with new OROV isolate AM0088.

HeLa cells were infected with OROV AM0088 (MOI 0.5). Cells were probed with AlexaFluor (AF)568 conjugated OROV nanobodies against N (NpE3) or Gc Spike (SpE8), with a polyclonal antibody against OROV and with an antibody against TGN46, plus appropriate secondary antibodies. Nuclei are stained with DAPI (blue). Scale bar = 10 µm.
